# Anti-IL5/IL-5 receptor therapies for eosinophilic granulomatosis with polyangiitis: an updated Systematic Review

**DOI:** 10.3389/fimmu.2025.1587158

**Published:** 2025-07-22

**Authors:** Matteo Lazzeroni, Valeria Longoni, Paolo Schiavo, Emanuele Bizzi, Antonio Brucato, Giulia Gramellini, Marco Borin, Alberto Dragonetti, Michele Gaffuri, Mario Lentini, Antonino Maniaci, Angela Mauro, Antonio Gidaro, Jan Schroeder, Pasquale Capaccio

**Affiliations:** ^1^ Department of Biomedical, Surgical and Dental Sciences, University of Milan, Milan, Italy; ^2^ Department of Otorhinolaryngology & Head and Neck Surgery, Fatebenefratelli Hospital, ASST Fatebenefratelli Sacco, Milan, Italy; ^3^ Department of Clinical Sciences and Community Health, University of Milan, Milan, Italy; ^4^ Internal Medicine Department, Fatebenefratelli Hospital, ASST Fatebenefratelli Sacco, Milan, Italy; ^5^ Department of Biomedical and Clinical Sciences, University of Milan, Fatebenefratelli Hospital, Milan, Italy; ^6^ Department of Otorhinolaryngology & Head and Neck Surgery, ASST Grande Ospedale Metropolitano Niguarda, Milan, Italy; ^7^ Department of Otolaryngology-Head and Neck Surgery, Fondazione IRCCS Ca’ Granda Ospedale Maggiore Policlinico, Milan, Italy; ^8^ Department of Otorhynolaringoiatry, ASPRagusa-Hospital Giovanni Paolo II, Ragusa, Italy; ^9^ Department of Medicine and Surgery, University of Enna Kore, Enna, Italy; ^10^ Department of Pediatrics, Service of Pediatric Rheumatology, Fatebenefratelli Hospital, Milano, Italy; ^11^ Internal Medicine Department, Sacco Hospital, ASST Fatebenefratelli Sacco, Milan, Italy; ^12^ Clinical Immunology, ASST Grande Ospedale Metropolitano Niguarda, Milan, Italy

**Keywords:** EGPA, IL5, eosinophil, mepolizumab, benralizumab

## Abstract

**Introduction:**

Eosinophilic Granulomatosis with Polyangiitis (EGPA) is a rare necrotizing vasculitis characterized by eosinophilic inflammation that was traditionally treated with corticosteroids associated with other immunosuppressants. Over the last years different biological therapies targeting IL-5/IL-5 receptor have become available and have been employed to tackle this challenging condition. Aim of the present study is to synthesis the evidence on the clinical presentation of this disease and on the efficacy of the newly available therapeutic strategies.

**Methods:**

In June 2024 PubMed, Embase and the Cochrane library were searched for studies reporting on EGPA patients being treated by means of different anti IL-5 or anti eosinophils biological therapies. Risk of bias was assessed through the ROBINS-I and RoB2 tools according to study design. Proportion meta-analysis was employed to synthetize data on EGPA clinical manifestations, while data on treatment outcomes was analyzed descriptively due to the high heterogeneity.

**Results:**

The present systematic review included 25 studies on a total of 1131 patients. Asthma was present in 99.2% of the patients, Sinonasal involvement in 87.0% and ANCA positivity in 22.8%. The explored treatments consisted in Benralizumab 30 mg every 4 weeks, Mepolizumab 100 mg or 300 mg every 4 weeks and Reslizumab 3mg/Kg every 4 weeks. All the anti-IL-5/IL-5 receptor molecules proved efficacious in remission control and corticosteroid tapering.

**Conclusion:**

The available data strongly suggests integrating anti IL-5/IL-5 receptor therapies into EGPA treatment strategies, to enhance patients’ outcomes and reduce the long term side effects of prolonged corticosteroid therapy.

## Introduction

Eosinophilic Granulomatosis with Polyangiitis (EGPA), formerly known as Churg Strauss syndrome, is a rare form of necrotizing vasculitis with extravascular granulomas occurring in patients with asthma and tissue eosinophilia (1). Clinically, EGPA typically presents with asthma, peripheral eosinophilia, and sinonasal involvement, such as chronic rhinosinusitis with nasal polyposis (CRSwNP). However, this condition is a multi-organ disease with a broad spectrum of clinical manifestations, leading to significant heterogeneity in presentation and severity. The pathogenesis of EGPA is still not fully understood, even if there is evidence suggesting the involvement of both environmental and genetic factors (2, 3). Organ damage in EGPA patients can occur with two different mechanisms: as a consequence of either vasculitis leading to ischemic effects and inflammation, which is prominent in myeloperoxidase anti-neutrophil cytoplasmic antibodies (MPO-ANCA)-positive patients, or either eosinophil-associated vascular occlusion leading to ischemia and eosinophil-associated tissue damage, which is frequent in MPO-ANCA-negative patients (4). A clear distinction between vasculitis driven organ damage and eosinophilic mediated damage is not always possible. Only tissue biopsies allow to assess weather vasculitis is present or not and eosinophilic inflammation can trigger the vasculitis process and disease progression (3, 5).

EGPA may arise at any age but is more frequently diagnosed in adults. Indeed, due to its heterogeneous presentations and overlaps with other conditions, the diagnosis of EGPA is often delayed. Both remission induction and remission maintenance of disease activity traditionally require corticosteroids, the tapering of which is often challenging. Therefore, patients are typically treated with prolonged and excessive doses of corticosteroids, which, while controlling effectively the systemic inflammation, can lead to serious metabolic, cardiovascular, osteoporotic and infectious side effects (5). Other immunosuppressive agents such as cyclophosphamide, rituximab, mycophenolate, azathioprine and methotrexate can also be employed in EGPA therapy, especially for preventing disease relapses during OCS therapy. However, their efficacy is still debated and controversial, and no clear indication on which therapy is to be used was released at international level (3).

The recent development of humanized monoclonal antibodies targeting IL-5 or IL-5 receptor (IL-5R) marked a significant breakthrough in the treatment of EGPA, revolutionizing its management and achieving disease control while progressively reducing OCS intake, as shown in both phase III trials (6, 7) and real-life settings (8, 9). Although some systematic reviews (10, 11) have investigated the demographic and clinical characteristics of EGPA patients, previous studies (12, 13) on biological therapies directed against the IL-5 pathway have not considered all available molecules together but have analyzed them individually or have focused on specific combination of treatments. The present work aims to fill these gaps, by providing an up to date and comprehensive synthesis of the evidence on the topic.

## Material and methods

This systematic review is written in accordance to the guidelines of the Cochrane Handbook for Systematic Reviews of Interventions (14) and followed the Preferred Reporting Items for Systematic reviews and Meta-Analysis (PRISMA) 2020 statement (15).

### Eligibility criteria

Inclusion in the present systematic review was restricted to studies that met all the following eligibility criteria: 1) Randomized or observational studies, 2) on a population of a minimum of 5 patients with EGPA, 3) receiving anti IL-5/IL-5R therapies. We excluded studies that did not report any clinical outcome. The cut-off of 5 patients was chosen to mitigate publication bias.

### Search strategy

In June 2024 PubMed, Embase and the Cochrane library were consulted with combinations of the following search terms: Churg-Strauss Syndrome, Churg-Strauss, Churg Strauss, EGPA, Eosinophilic Granulomatosis with Polyangiitis, Mepolizumab, Benralizumab, anti-IL-5 biologics, anti-IL-5. The full search strategies employed in the searched databases are reported in **Supplementary File 1**. No language restrictions were applied to be as complete as possible. Two authors (ML and VL) independently performed the abstract and full text screening of the retrieved articles through Rayyan (16), a free software specifically designed for screening of abstracts, titles and full texts through a process of semi-automation. Disagreements in any phase were resolved through discussion. If there was an overlap amongst studies populations, only the largest cohort was included in the present study.

### Data extraction

Two reviewers (ML and VL) independently extracted relevant data from the selected articles: name of the first author and country of origin, year of publication, study design, sample size, age and sex of the enrolled patients. Data on clinical manifestations, signs, and organ damage at the time of disease diagnosis were collected, such as asthma, neuropathy, pulmonary infiltrates, sinonasal abnormalities, cardiac/renal/dermatological involvement, and ANCA positivity at baseline.

Pre-treatment data included, when described, the blood eosinophil count (BEC) and oral corticosteroid dose (OCS). For each study, treatment outcomes were recorded. These outcomes were diverse and included measures of disease remission, defined according to the European League Against Rheumatism (EULAR) as a Birmingham Vasculitis Activity Score (BVAS) of 0 and an OCS dose ≤7.5 mg/day (17). Changes in the proportion of patients achieving remission over the study periods were analyzed, as well as variations in BEC, OCS dose and disease activity scores (BVAS). Additional outcomes were changes in the Asthma Control Test (ACT) scores, Sinonasal Outcome Test-22 (SNOT-22) scores, forced expiratory volume in one second (FEV1), and time to first disease relapse. Data extraction of numerical data from graphs was performed through WebPlotDigitizer.

### Data synthesis

Proportion meta-analysis was used to analyze the demographic characteristics of the included EGPA patients effectively. Proportion meta-analysis is a statistical method usually employed to synthetize evidence, such as proportions or prevalence, from single group studies on rare conditions. A random effects model was chosen, as well as the Freeman–Tukey double-arcsine transformation. We assessed heterogeneity with I^2^ statistics and Cochrane Q test; p-values < 0.10 and I^2^ > 40% were considered significant for heterogeneity. R software (version 4.4.0, 2024-04-24) was used for the statistical analysis.

Due to the highly variable study designs, the lack of comparative studies, the highly heterogeneous follow up times and reported outcomes, meta-analytic synthesis was not deemed applicable to changes in remission rates and disease activity. Therefore, therapeutic outcomes were analyzed descriptively. Continuous variables are reported as means and standard deviations when normally distributed, or as medians and interquartile range when non-normally distributed. Categorical variables are reported as absolute frequencies and valid percentages.

### Quality assessment

The risk of bias assessment of the included studies was performed with the Risk Of Bias In Non randomized Studies of Interventions (ROBINS-I) tool (18) in case of observational, cohort studies or case series. While risk of bias for randomized studies was assessed through the Cochrane Collaboration’s tool for assessing risk of bias in randomized trials (RoB-2) (19). Two authors (ML and VL) independently performed the quality assessment, resolving conflicts through discussion.

## Results

### Study selection

**Figure 1:**
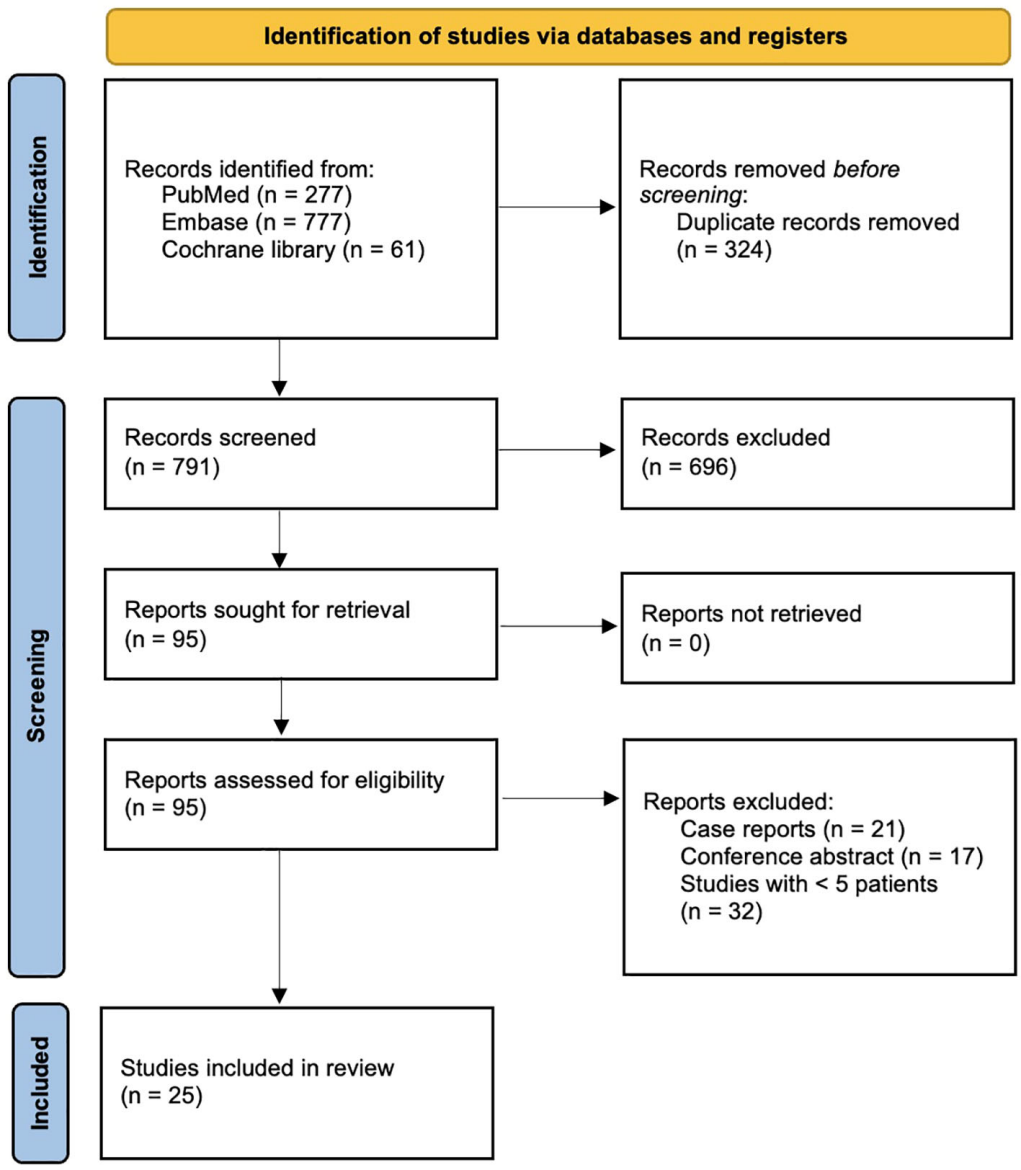
PRISMA flowchart of the study selection process for the present systematic review and meta-analysis.

**Figure 1** shows the PRISMA flowchart for the article selection process. A total of 791 records were retrieved from PubMed, Embase and the Cochrane library. After title and abstract screening, 95 studies remained and were assessed in their full text. Eventually, 25 studies (4, 6–9, 20–39) were included in the present systematic review. Two were randomized controlled trials (6, 7) and 23 observational studies (4, 8, 9, 20–39).

### Baseline characteristics

The present systematic review comprises a total of 1131 patients, of which 44% were male and 56% were female. These patients were mostly adult, being their age relatively homogeneous across studies. General baseline characteristics of the patients are presented in **Table 1**. The blood eosinophil count, however, showed significant variability: Wechsler et al. (6) reported mean BEC values of mean 177 ± 1.29, while Bostan et al. (20) a median BEC of 1000 (700-1800). The use of OCS among the included patients before starting their anti-IL-5/IL-5R therapies was consistently high, with values ranging from 8.75 (5-15) to 19.5 (5-40) mg.

**Table 1:** baseline characteristics, each cohort of patients with EGPA of the included articles is described separately.

Study	Number of patients	BVAS>0 (No.)	Intervention	Age (years)	Male	Female	BEC*	OCS (mg/d)
Wechsler et al., 2017 (6)	68	37	Mepolizumab 300 mg 4w	49.0 ± 12.0	26 (38%)	42 (62%)	177.0 ± 1.29	12.0 (7.5-40.0)
Wechsler et al., 2024 (7)	70	33	Mepolizumab 300 mg 4w	52.7 ± 14.4	25 (36%)	45 (64%)	384.9 ± 563.6	10.0 (7.5-40.0)
	70	34	Benralizumab 30 mg 4w	52.0 ± 13.10	31 (44%)	39 (55%)	306.0 ± 225.1	10.0 (5.0-30.0)
Bettiol et al., 2022 (8)	158	144	Mepolizumab 100 mg 4w	48.7 (37.9–57.5)	70 (44%)	88 (55%)	700.0 (200.0-1080.0)	10.0 (5.0-20.0)
	33	31	Mepolizumab 300 mg 4w	49.2 (39.8–53.4)	11 (33%)	22 (66%)	440.0 (200.0-910.0)	10.0 (5.0-22.5)
Bettiol et al., 2023 (9)	121		Benralizumab 30 mg 4w	54.1 (44.2–62.2)	57 (47%)	64 (53%)		
Bostan et al., 2023 (20)	11		Mepolizumab 300 mg 4w	48.0 (36.0-54.0)	10 (90%)	1 (10%)	1000.0 (700.0-1800.0)	16.0 (8.0-16.0)
Canzian et al., 2021 (21)	51		Mepolizumab	46.0 (38.0–55.0)	21 (41%)	30 (50%)	770.0 (342.0-1135.0)	10.0 (7.5-25.0)
Cottu et al., 2023 (22)	68		Benralizumab 30 mg 4wx3 => 8w	50.0 (39.0–63.0)	39 (57%)	29 (43%)	340.0 (87.0-875.0)	10.0 (6.0-15.0)
Desaintjean et al., 2024 (23)	26		Mepolizumab or Benralizumab	49.5 (21.0–77.0)	12 (46%)	14 (54%)		
Detoraki et al., 2021 (24)	8		Mepolizumab 100 mg 4w	55.8 ± 13.13	6 (75%)	2 (25%)	2384.0 ± 2209.0	16.7 ± 9.0
Guntur et al., 2021 (25)	10	10	Benralizumab 30 mg	47.0 ± 17.0	5 (50%)	5 (50%)	350.0 ± 311.0	15.0 (5.0-20.0)
Ishii et al., 2023 (26)	118		Mepolizumab 300 mg 4w	61.9 ± 13.2	53 (45%)	65 (55%)		8.6 ± 7.7
Kim et al., 2010 (27)	7		Mepolizumab 750 mg 4w	45.0 (28-62)	2 (29%)	5 (71%)		12.9 (10-20)
Manka et al., 2021 (28)	10	10	Reslizumab 3 mg/kg	45.5 ± 15.48	6 (60%)	4 (40%)	133.3 ± 141.4	19.5 (5-40)
Masumoto et al., 2023 (29)	43		Mepolizumab	59.9 ± 14.1	16 (37%)	27 (63%)		42.9 ± 11.7
Matsuno 2014 (30)	20		Mepolizumab 300 mg 4w	60.6 ± 9.52	3 (15%)	17 (85%)		8.9 ± 5.0
Nakamura et al., 2022 (31)	13	5	Mepolizumab 100 mg 4w	59.0 (52.0 - 66.5)	4 (30%)	9 (70%)	370.0 (200.0-880.0)	5.0 (4.5-8.3)
Nanzer et al., 2024 (32)	70	54	Benralizumab 30 mg	49.4 ± 14.3	34 (49%)	36 (51%)		13.1 (10.5)
Nolasko et al., 2023 (33)	26		Benralizumab 30 mg	49.2 ± 12.9	12 (46%)	14 (54%)	890.0 (506.0-1800.0)	10 (5-15)
	23		Mepolizumab 300 mg 4w	51.5 ± 9.8	6 (26%)	17 (74%)	705.0 (415.0-1409.0)	12.5 (5-25)
Padoan et al., 2020 (34)	5		Benralizumab 30 mg	42.0 (32.5-55.5)	1 (20%)	4 (80%)		12.5 (11.3-15.0)
Ramirez et al., 2022 (35)	14		Mepolizumab 300 mg 4w	51 (49–56)	4 (29%)	10 (71%)		8.8 (5.0-15.0)
Ríos-Garcés et al., 2022 (4)	11		Mepolizumab	48.6 ± 14.7	6 (55%)	5 (45%)		11.4 (5.0-22.5)
Ueno et al., 2022 (36)	7		Mepolizumab 300 mg 4w	74.0 (63.0, 83.0)	3 (42%)	4 (58%)		
Vultaggio et al., 2020 (37)	18		Mepolizumab 100 mg 4w	57.0 ± 8.7	6 (33%)	12 (67%)		8.1 ± 1.1
Yamane at al. 2023 (38)	27		Mepolizumab	59.0 (53.5-70.0)	15 (55%)	12 (44%)	431.0 (332.0-670.0)	5.0 (3.1-7.5)
Özdel Öztürk et al., 2022 (39)	25		Mepolizumab 100 mg 4w	47.0 (23.0-76.0)	7 (28%)	18 (72%)		

BEC, blood eosinophilic count; OCS, daily dose of oral corticosteroid; No=number, *: Absolute eosinophil count per cubic millimeter. Continous variables are reported as means ± standard deviation or medians (interquartile range).

### Disease manifestations

**Figure 2:**
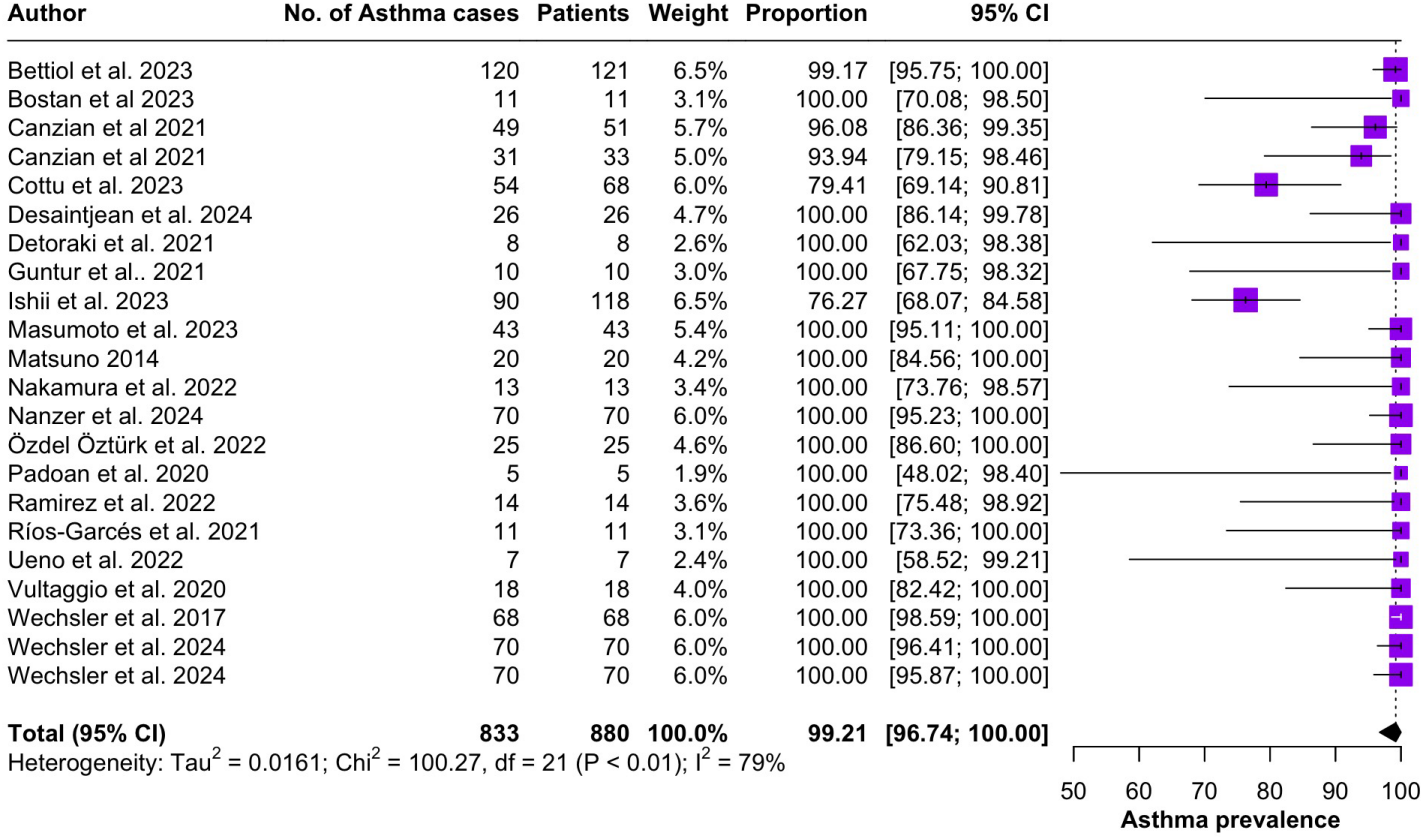
Forest plot reporting on the overall prevalence of asthma among the included patients.

**Figure 3:**
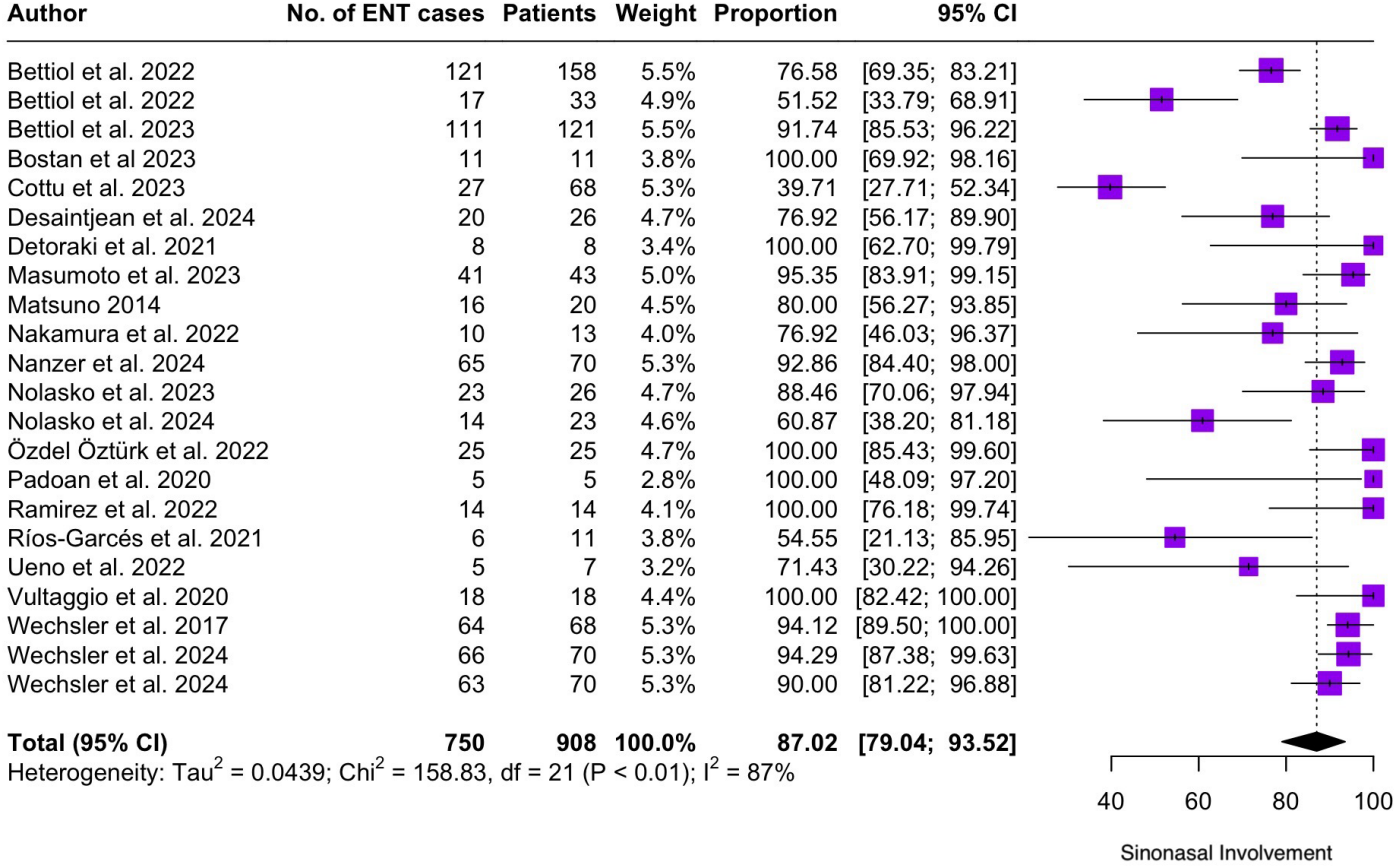
Forest plot reporting on the overall prevalence of sinonasal involvement among the included patients.

**Figure 4:**
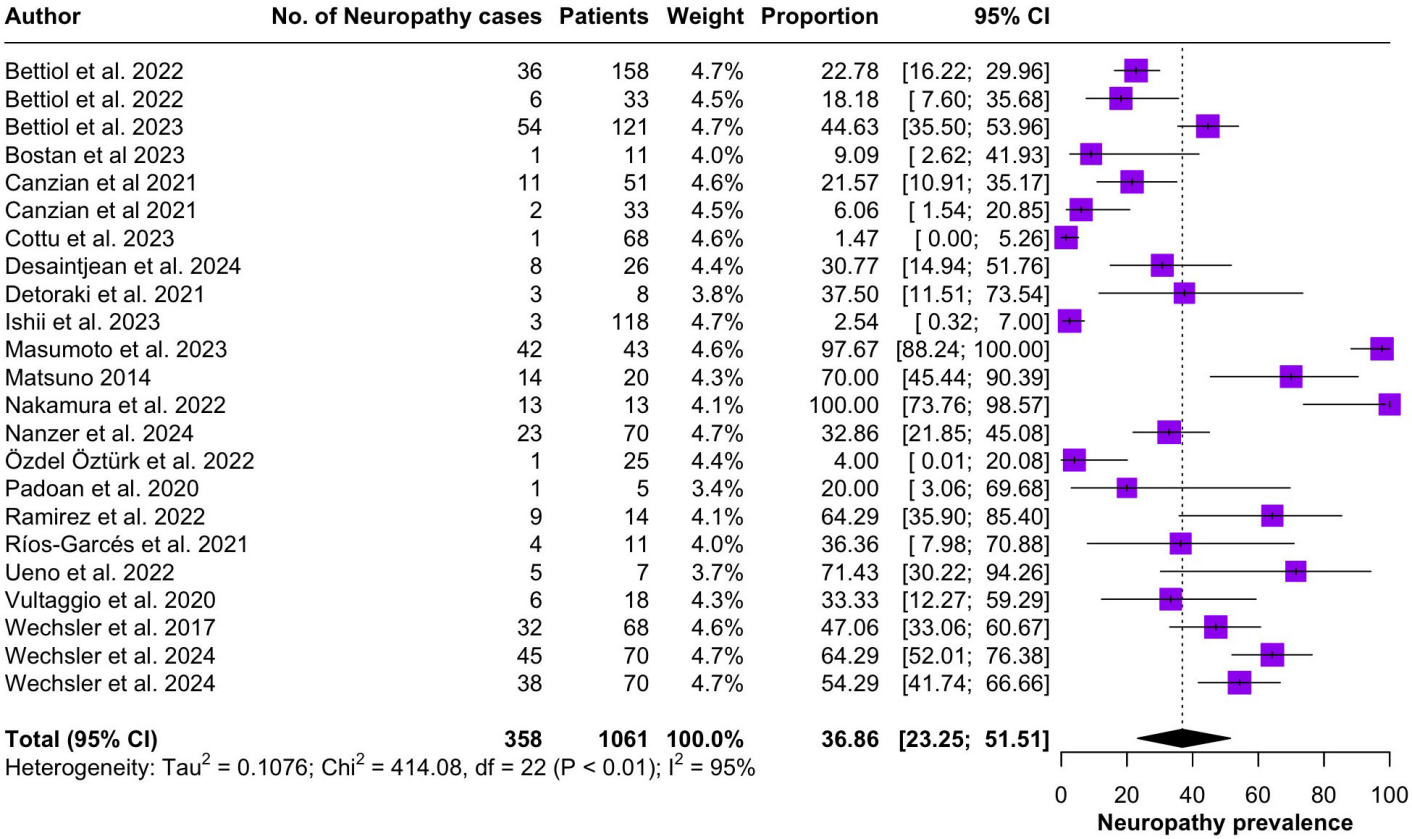
Forest plot reporting on the overall prevalence of neuropathy among the included patients.

**Figure 5:**
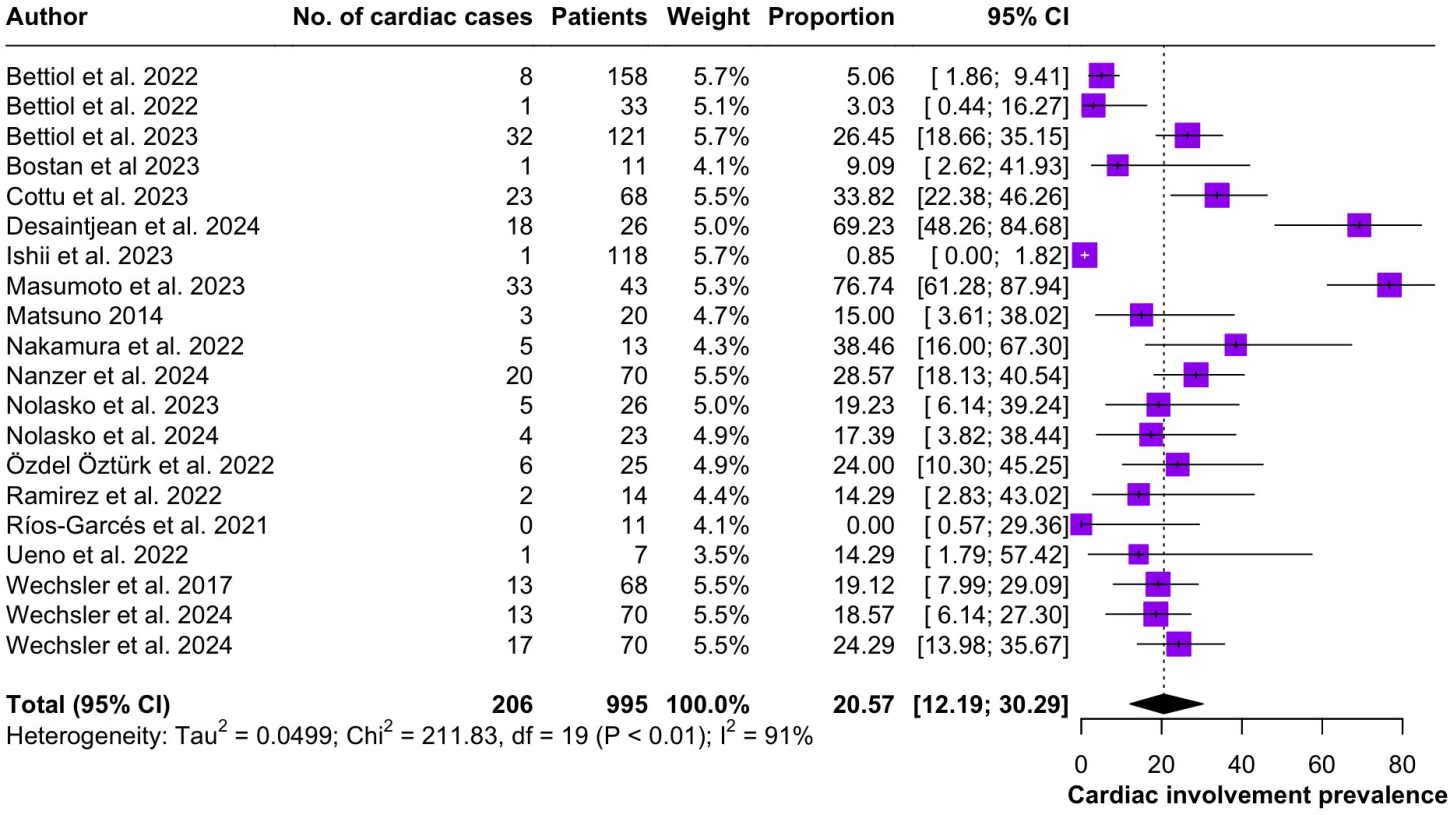
Forest plot reporting on the overall prevalence of cardiac involvement among the included patients.

**Figure 6:**
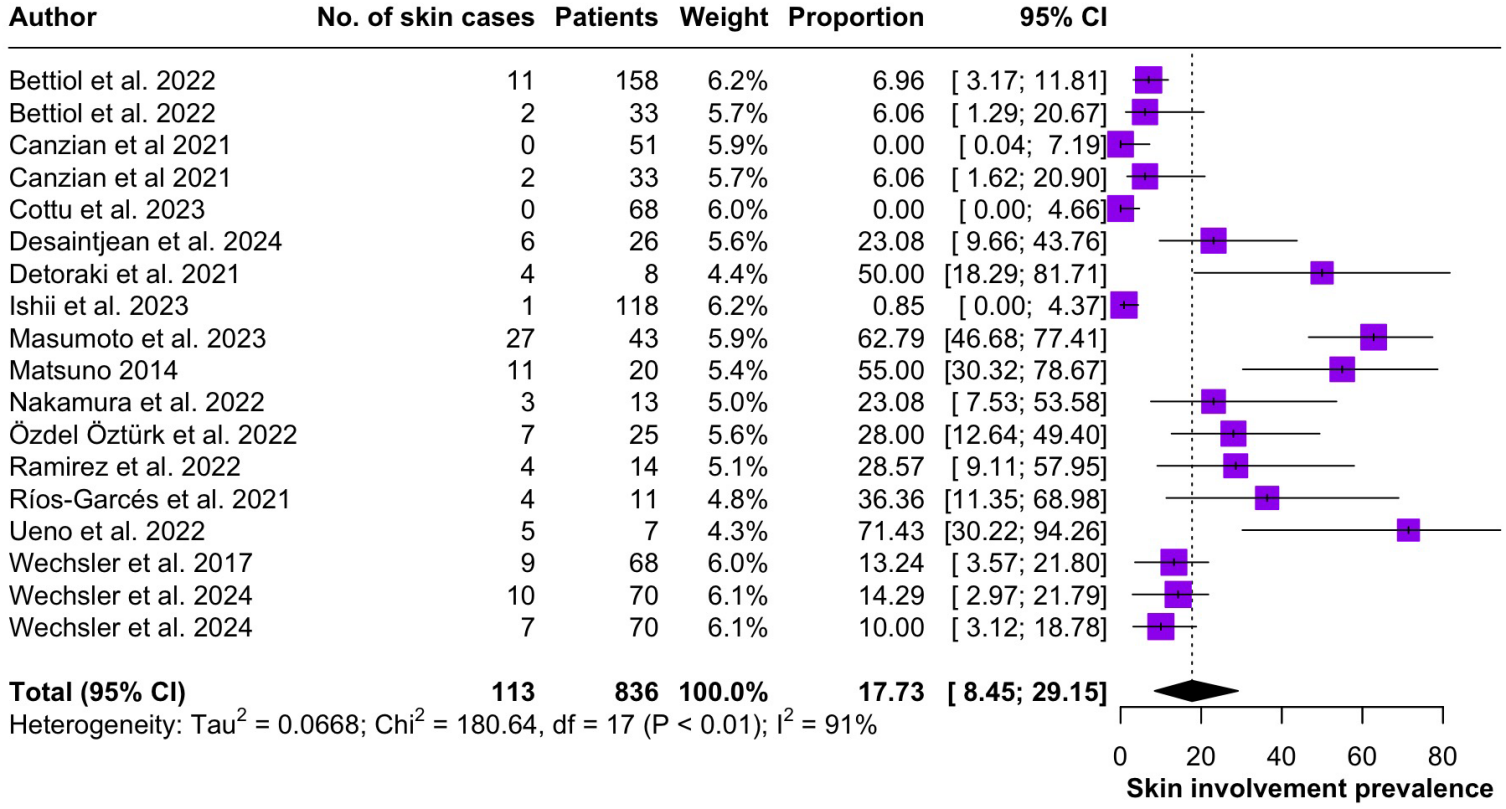
Forest plot reporting on the overall prevalence of cutaneous involvement among the included patients.

**Figure 7:**
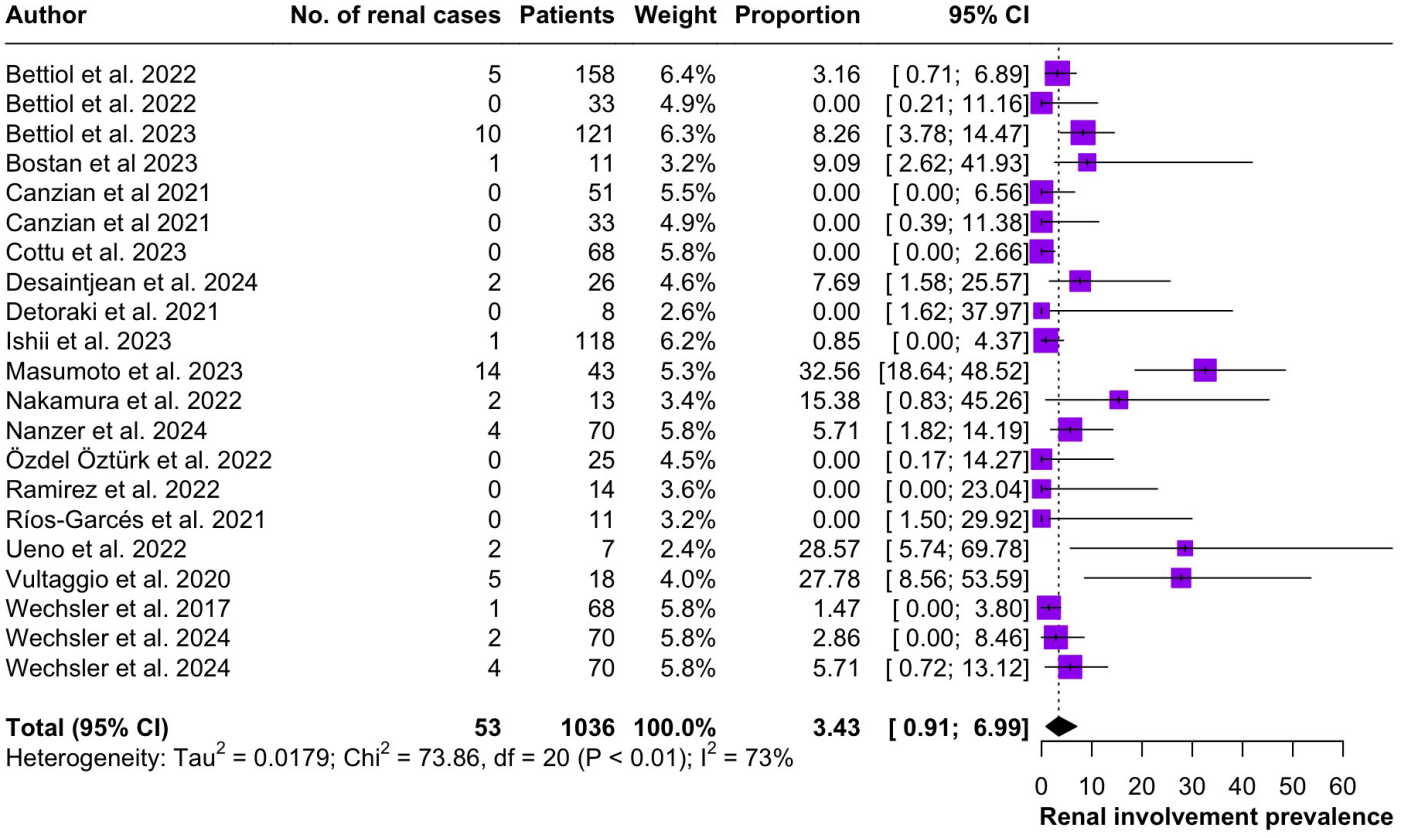
Forest plot reporting on the overall prevalence of renal involvement among the included patients.

**Figure 8:**
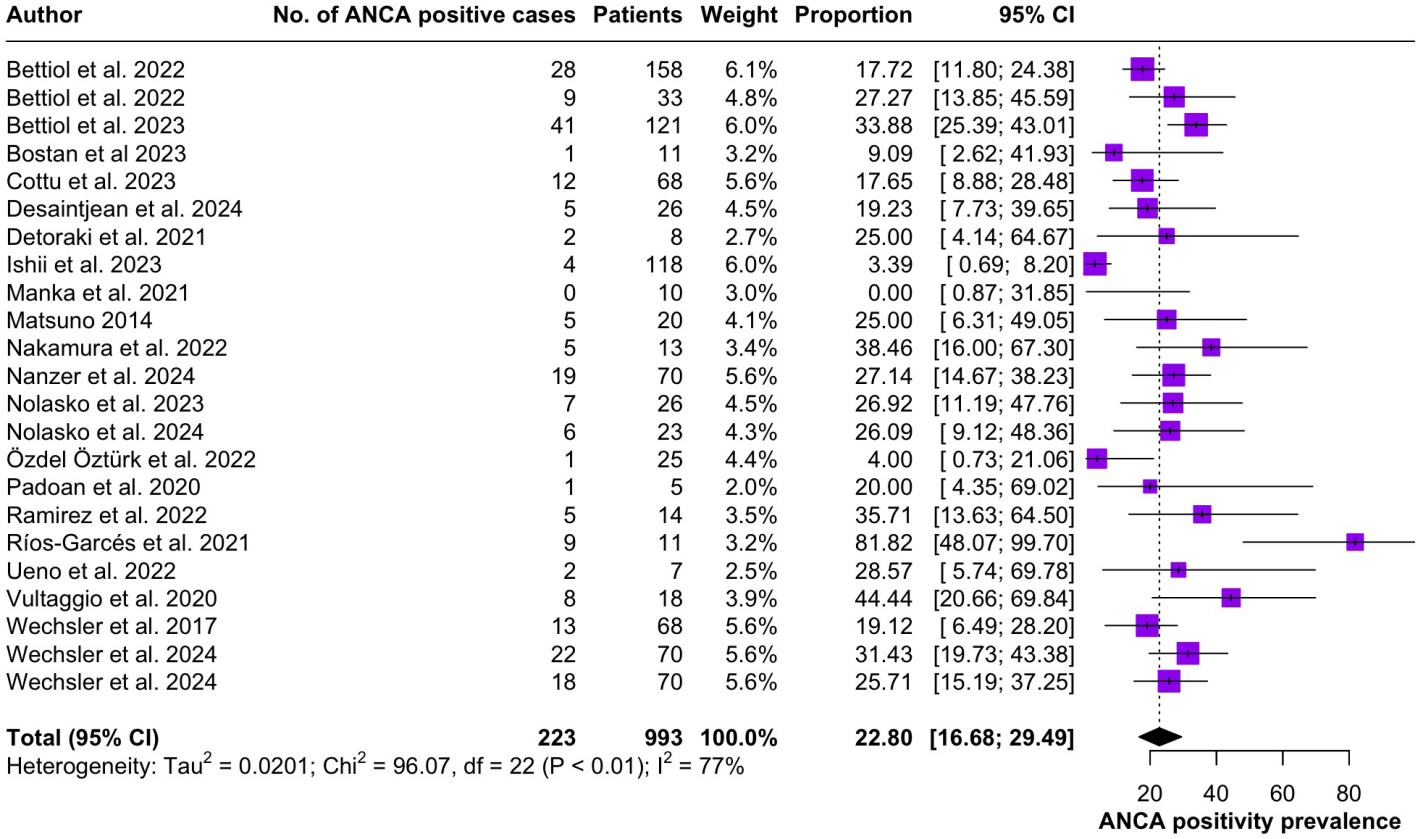
Forest plot reporting on the overall prevalence of ANCA positivity among the included patients.

Regarding the clinical manifestations of EGPA, asthma was the most consistent feature with a pooled prevalence of 99.2% (95% CI 96.7-100.0, I^2^ = 79%) (**Figure 2**). Sinonasal involvement was also frequent, as marked by its pooled prevalence of 87.0% (95% CI 79.0-93.5, I^2^ = 87%)(**Figure 3**). Neuropathy was present in 36.9% of the patients (95% CI 23.3-51.5%, I^2^ = 95%), the I^2^ showing great heterogeneity between studies (**Figure 4**). Cardiac and cutaneous involvement were less commonly observed, with a pooled prevalence of 20.6% (95% CI 12.2-30.3%, I^2^ = 91%) and 17.7% (95% 8.5-29.2, I^2^ = 91%) respectively (**Figures 5**, **6**). Renal involvement was even more rare, with a pooled prevalence of 3.4% (95% CI 0.9-7.0%, I^2^ = 73%) (**Figure 7**). Lastly, ANCA positivity was present in 22.8% of the patients (95% CI 16.7-29.5, I^2^ = 77%) (**Figure 8**).

### Treatment

Among the included studies mepolizumab was the most frequently used anti IL-5 biologic in EGPA patients. The effects of different therapeutic doses were investigated, with several studies (6–8, 20, 26, 30, 33, 35, 36) evaluating its efficacy at a dose of 300 mg every 4 weeks, while others (8, 24, 31, 37) at 100 mg every 4 weeks. Notably, Kim et al. (27) treated their patients with 750 mg administered every 4 weeks. Benralizumab was also assessed in different studies, mostly prescribing it at the dosage of 30 mg every 4 weeks (7, 9, 25, 32–34). Cottu et al. (22) evaluated a regimen of 30 mg every 4 weeks for 3 doses, followed by administration every 8 weeks. Lastly, only one of the included articles (28) investigated the efficacy of Reslizumab (3 mg/kg).

The included studies followed different protocols for the timing of anti-IL-5/IL-5R therapy initiation. Bettiol et al. (8) and Cottu et al. (22) included patients who were already on long-term corticosteroid therapy. Similarly, the MIRRA and MANDARA trials (6, 7) evaluated IL-5/IL-5R antagonists as add-on agents in patients with relapsing or corticosteroid-dependent EGPA, rather than exploring their effect as induction therapy. However, studies such the ones of Nolasco et al. (33) and Ríos-Garcés et al. (4) do not indicate how long after being diagnosed with EGPA their patients were prescribed with anti-IL-5/IL-5R therapies.

The retrieved outcomes are presented in **Supplementary File 1**. Amongst this was the induction of disease remission according to the definition proposed by the European League against Rheumatism (17). Both mepolizumab and benralizumab showed promising results in this regard in all the 6 studies that included the information (6–9, 32, 33). The anti-IL-5/IL-5R therapies were also rapidly efficacious in controlling circulating eosinophils, being able to significantly reduce BEC already after the first 12 weeks of treatment (8, 9, 20, 22, 31, 34). Notably, Bettiol et al. (9), Cottu et al. (22) and Nolasko et al. (33) showed how benralizumab managed to reduce BECs to zero in their cohorts of patients. All the included studies showed how anti IL-5/IL-5R biological therapies are efficacious in obtaining sustained control of circulating eosinophils at 48 weeks (8, 9, 20, 22, 24, 31, 33, 34, 37, 39).

All the retrieved articles showed a significant reduction in OCS use by EGPA patients after all the anti-IL-5/IL-5R biological therapies. Bostan et al. (20) in particular showed how their patients were able to stop taking OCS, going from mean dosage of 16 (8-16) to 0 (0-4), similarly Nolasko et al. (33) cohort of patients taking Mepolizumab 300 mg 4w went from 12 (5-25) to 0 (0-5).

Lastly, the anti-IL-5/IL-5R biologics showed promising results in both rhinologic and pneumological domains. SNOT22 scores significantly decreased in EGPA patients after 24 weeks of therapy and continued to get lower until the end of the studies follow-up time at 48 weeks (20, 24, 37). The airflow measurements (FEV1) also homogeneously, progressively continued to improve in the retrieved studies (9, 20, 27, 32–34).

### Quality assessment

According to the RoB-2 tool the risk of bias of the 2 randomized studies (6, 7) was deemed as “Low”, while According to the ROBINS-I tool the overall risk of bias of the 23 articles was “moderate” for 9 articles (8, 9, 21, 25, 31–33, 38, 39), “serious” for 3 (22, 26, 27) and “critical” for 11 (4, 23, 24, 28–30, 34–37).

## Discussion

The present work represents the largest systematic review on EGPA, comprising 25 studies and a total of 1131 patients. It provides an updated overview of the demographic characteristics, clinical manifestations and therapeutic options for this rare condition.

Regarding organ involvement, upper and lower respiratory tracts are the most frequently affected sites (3). Indeed, our findings confirm that nearly all included patients had asthma (99.2%; 95% CI 96.7-100.0) and that the vast majority suffered from sinonasal involvement (87.0%; 95% CI 79.0-93.5). CRSwNP associated with late onset asthma in a patient with blood eosinophilia, undergoing chronic OCS treatment should be considered an important red flag for a possible EGPA (40). However, beyond CRSwNP, EGPA can manifest with a variety of ENT conditions, such as allergic rhinitis and hearing loss/otitis media. The latter has a prevalence that ranges between 10-20% according to the literature (41, 42) and typically falls under the category of eosinophilic otitis media: a type 2 inflammatory disease that is refractory to most conventional surgical and medical treatments (43).

Peripheral nerve involvement was observed in 36.9% of the patients included in the present systematic review. Neurological manifestations of EGPA most commonly present as multiple mononeuropathies, distal polyneuropathy or lumbar radiculopathy (3). The etiopathogenesis of neural involvement is thought to be directly linked to eosinophilic damage (44), as eosinophils play a pivoting role in driving disease progress. While EGPA has been classified as an anti-neutrophil cytoplasm antibody (ANCA)-associated vasculitis, ANCA positivity is not a consistent finding in these patients. Cung et al. (45) reported that only 40% of EGPA patients produce detectable ANCA and our results indicate an even lower prevalence of 22.8% (95% CI 16.7-29.5).

EGPA treatment aims both at controlling the systemic vasculitis activity and progressively reduce corticosteroid dependence (46). OCS, either alone or in association with cyclophosphamide or rituximab, remains the cornerstone for remission induction in EGPA. Before the advent of anti-IL-5/IL-5R therapies, other immunosuppressants such as azathioprine, methotrexate and mycophenolate mofetil were employed for remission maintenance (47).

The MIRRA trial showed that mepolizumab added to OCS, with or without conventional immunosuppressive drugs for induction, granted higher rates of sustained remission and OCS sparing in EGPA (6). Lastly, the MANDARA study later demonstrated the non-inferiority of benralizumab to mepolizumab in the induction of EGPA remission (7).

Our review further highlights the efficacy of anti-IL-5/IL-5R therapies in maintaining disease remission and OCS tapering, showing significant reduction OCS daily doses, as well as improved respiratory outcomes and marked reduction of circulating eosinophils throughout the follow up period.

Despite the extremely promising results reported in the registration studies of Mepolizumab and Benralizumab in Patients affected by EGPA, many aspects of therapy with anti-IL-5/IL-5R drugs still remain to be clarified.

First of all, there is no uniformity at an international level regarding the correct strategy for inducing remission in patients with EGPA; some Authors have proposed models of use of Rituximab associated with variable doses of OCS, other Authors have proposed the association of OCS with immunosuppressants of various nature, variable depending on the clinical characteristics of acute presentation. This is reflected in an uncertainty that often finds an answer in the experience and approach of the individual Center, therefore not contributing to guaranteeing uniformity of data in the evaluation of the different initial approaches.

In addition to this, EGPA itself can present with two different serological subsets, p-ANCA+ and p-ANCA-. As noted in a recent study by Piga et al. (48), therapy with anti-IL-5/IL-5R drugs may give rise to different results depending on the serological status of the Patient, underlining a possible pathogenic role of p-ANCA, which is still the subject of heated debate.

Furthermore, in the same paper the Authors emphasize how the different approaches used in the induction of remission can lead, both in p-ANCA- and p-ANCA+ patients, to different results, taking into account that p-ANCA may play a pathogenetic role once the eosinophilic response is ablated, which both drugs are demonstrably able to produce.

Both registration studies did not emphasize this possible serological difference nor did they evaluate any differences in the frequency of exacerbations or in the clinical manifestation of exacerbations, whose characteristics of organ involvement would be extremely important to attribute a possible pathogenic role to p-ANCA if the Patients who were carriers were more prone to developing relapses.

These data could be derived from phase IV studies if the right attention was paid both to the methods of induction of remission and to any differences in frequency and clinical characteristics of relapses.

Recent studies on large cohorts of patients are slowly highlighting peculiar aspects regarding the efficacy of Mepolizumab in maintaining remission and the retention rate of the drug in patients affected by EGPA (49–53), but to fully understand what is the best approach for inducing remission and how much the co-presence of p-ANCA affects the development of relapses, further studies on even larger case studies are needed, with targeted sub-analyses on the different subgroups of patients.

A recent *post-hoc* analysis has established that the need for high doses of OCS at baseline may represent a predictive factor of lower response to Mepolizumab, regardless of the type of immunosuppressant used in association with at baseline (54). These data seem to underline only that Mepolizumab may be less effective in cases where the disease has proven more aggressive and has required higher doses of OCS, but does not clearly establish whether the presence of p-ANCA represents a negative predictive factor nor does it specify the efficacy of the type of immunosuppressant used, together with OCS, in inducing remission before the use of Mepolizumab.

International multicenter studies are necessary, given the relative rarity of the disease, which allow case studies numerically sufficient to draw conclusions with statistical validity and able to direct clinicians to more targeted and informed therapeutic choices.

The present study has several limitations. Firstly, the included studies varied greatly in terms of study designs, treatments and outcomes. Due to the rarity of EGPA, there is a lack of comparative studies evaluating the success rates of different treatments and their combinations. Moreover, outcomes of different therapeutic strategies for EGPA were also reported heterogeneously between studies, some including organ specific parameters such as FEV1 and SNOT22 scores (24, 34), others focusing on the number of patients achieving disease remission (6–8). This great inconsistency prevented us from synthesizing available evidence through meta-analytic analysis. Furthermore, as remarked by our results, most included records are non-randomized, observational cohort studies that are at severe risk of bias, an issue that we have tackled through a rigorous quality assessment as indicated by the Cochrane Handbook for Systematic Reviews of Interventions (14). Studies with a high risk of bias are common when dealing with rare conditions, such as EGPA, since most records available on the topic will be simple case series with methodological issues such as the lack of a control group, incomplete outcome reporting, variability in definitions of treatment response. The Cochrane Handbook for Systematic Reviews of Interventions (14) doesn’t suggest to exclude these studies from systematic reviews, but rather to acknowledge their limitations in order to interpret the pooled findings with caution. Another limitation of the present study is that the results on clinical manifestations only apply to individuals receiving anti-IL-5/IL-5 receptor medications, therefore, while evaluating these findings, selection bias should be taken into account. However, our pooled analysis’s prevalence of important disease characteristics (such as asthma and sinonasal involvement) matches that of larger EGPA cohorts, indicating that any selection bias might not significantly skew the clinical picture presented by the present meta-analysis. Lastly, due to this variability in study designs, the precise timing of anti-IL5/IL-5R therapy initiation could not be uniformly assessed across the included studies, highlighting the need for phase IV trials focusing on induction strategies.
