# Supplementary material for: Anti-IL5/IL-5 receptor therapies for eosinophilic granulomatosis with polyangiitis: an updated Systematic Review
Source: Front Immunol. 2025 Jul 22;16:1587158. doi: 10.3389/fimmu.2025.1587158 (PMC12321870; doi:10.3389/fimmu.2025.1587158)
Supplement: Supplementary file 1 [file DataSheet1.docx]

**Supplementary file 1**

- 1. Remission: table showing the remission rates of the patients included in the present systematic review after 12, 24, 36 and 48 weeks of therapy with anti IL5 biologics. Remission was defined according to the European League Against Rheumatism (EULAR) as a Birmingham Vasculitis Activity Score (BVAS) of 0 and an OCS dose ≤7.5 mg/day. *: cohort of patients treated with mepolizumab, **: cohort of patients treated with benralizumab

| **Study** | **treatment** | **12w** | **24w** | **36w** | **48w** |
| --- | --- | --- | --- | --- | --- |
| Wechsler et al. 2017 | * | 25 | 36 | 32 | 31 |
| Wechsler et al. 2024 | * | 9 | 31 | 40 | 48 |
|  | ** | 14 | 34 | 42 | 47 |
| Bettiol et al. 2022 | * | 19 | 35 |  | 38 |
|  | * | 6 | 9 |  | 11 |
| Bettiol et al. 2023 | ** | 15 | 25 |  | 32 |
| Nanzer et al. 2024 | ** |  |  |  | 47 |
| Nolasko et al. 2023 | ** | 0 | 6 |  | 11 |
|  | * | 2 | 7 |  | 8 |

- 1. BEC control: Table showing the blood eosinophil count trend over time after initiation of anti IL5 therapies. Data are expressed in cells/mm^3^ . *: cohort of patients treated with mepolizumab, **: cohort of patients treated with benralizumab

| **Study** | **treatment** | **0w** | **12w** | **24w** | **pr48w** |
| --- | --- | --- | --- | --- | --- |
| Bettiol et al. 2022 | * | 700 (200-1080) | 80 (40-160) | 80 (25-130) | 80 (20-125) |
|  | * | 200 (440-910) | 70 (40-120) | 60 (40-100) | 70 (30-100) |
| Bettiol et al. 2023 | ** | 535.0 (140.0–1000.0) | 0.0 (0.0-4.5) | 0.0 (0.0-2.5) | 0.0 (0.0-7.5) |
| Bostan et al 2023 | * | 1000 (700–1800) | / | 100 (0–200) | 100 |
| Cottu et al. 2023 | ** | 310 (70-690) | 0 (0-3 | 0 (0-0) | 0 (0-0) |
| Detoraki et al. 2021 | * | 2380 |  | 200 | 150 |
| Nakamura et al. 2022 | * | 370 (200–880) |  | 88 (23-151) | 50 (3–88) |
| Nolasko et al. 2023 | ** | 890 (506-1800) | 0 (0-0) | 0 (0-0) | 0 (0-0) |
|  | * | 705 (415-1409) | 100 (65-117) |  | 105 (45-125) |
| Padoan et al. 2020 | ** | 1200 (555 1495) |  | 0 (0 0) |  |
| Vultaggio et al. 2020 | * | 785 (454-1034) | 72 (57-84) | 74 (38-94) | 60 (25-88) |
| Özdel Öztürk et al. 2022 | * | 1,610 (153) |  | 102 (13) | 109 (34) |

- 1. OCS: Table showing the median (IQR) doses of oral corticosteroids taken by the included patients after the initiation of anti IL5 therapies. OCS doses are expressed in mg. *: cohort of patients treated with mepolizumab, **: cohort of patients treated with benralizumab

| **Study** | **treatment** | **0w** | **12w** | **24w** | **36w** | **48w** |
| --- | --- | --- | --- | --- | --- | --- |
| Wechsler et al. 2017 | * | 12.0 | 8.9 | 5.4 | 5.0 | 4.6 |
| Wechsler et al. 2024 | * | 10,9 | 7,8 | 5,4 | 3,9 | 3,1 |
|  | ** | 11,1 | 7,7 | 4,9 | 3,6 | 3 |
| Bettiol et al. 2022 | * | 10 (5-20) | 5 (3.5-10) | 5 (2-7.5) |  | 5 (0-5.5) |
|  | * | 10 (5-25) | 5 (4.5-7.5) | 5 (2.5-5.5) |  | 2,5 (0-5) |
| Bettiol et al. 2023 | ** | 10 (5-12) | 5 (3.5-8.5) | 5 (2.5-6.2) |  | 2,5 (0-5) |
| Bostan et al 2023 | * | 16 (8-16) | / | 4 (0–4) | / | 0 (0–4) |
| Canzian et al. 2021 | * | 10 (7.5–25) |  | 5 (3.75–7.5) |  | 3.9 (2.1–6.9 |
|  |  | 15 (10–20 |  | 11 (6.9–15) |  | 10 (6.5–15.5) |
| Cottu et al. 2023 | ** | 10.0 (6.8- 16.3) | 7.5 (5.0-12.5) | 6.0 (5.0-10.0) |  | 5.0 (0.0-5.5) |
| Desaintjean et al. 2024 | */** | 10 (7.6–20) |  | 9 (5–10) |  | 5 (2–10) |
| Detoraki et al. 2021 | * | 16.7 (13.5-19.9) |  | 6.8 (3.7-9.7) |  | 5.3 (1.8-8.8) |
| Guntur et al.. 2021 | ** |  |  |  |  |  |
| Ishii et al. 2023 | * | 6.9-8.6 |  | 2.5 |  | 2 |
| Kim et al. 2010 | * | 18.8 | 4.6 | 4.3 |  |  |
| Manka et al. 2021 |  | 19.5 |  |  |  |  |
| Nakamura et al. 2022 | * | 5.0 (4.5–8.3) |  |  |  | 2.0 (0.5–3.5) |
| Nanzer et al. 2024 | ** | 13.1 (10.5) |  |  |  | 2.4 (6.0) |
| Nolasko et al. 2023 | ** | 10 (5-15) | 5 (4-6) | 3 (3-5) |  | 2 (0-5) |
|  | * | 12 (5-25) | 5 (2-13) | 5 (2-8) |  | 0 (0-5) |
| Padoan et al. 2020 | ** | 12.5 (11.25-15) |  | 0 (0-3.12) |  |  |
| Ríos-Garcés et al. 2021 | * | 11.4 (5-22.5) |  |  |  |  |
| Ueno et al. 2022 | * | 30.0 (30.0, 40.0) |  | 5.0 (5.0, 10.0) |  |  |
| Özdel Öztürk et al. 2022 | * | 11.04 (1.72) |  | 3.65 (0.67) |  | 2.76 (0.76) |

- 1. SNOT22: outcomes of the SNOT22 tests of the included patients. *: cohort of patients treated with mepolizumab, **: cohort of patients treated with benralizumab

| **Study** | **treatment** | **0w** | **12w** | **24w** | **36w** | **48w** |
| --- | --- | --- | --- | --- | --- | --- |
| Bostan et al 2023 | * | 70 (53–82) | / | 19 (4–35) | / | 11 (6–40) |
| Detoraki et al. 2021 | * | 48 (40-57) |  | 25 (21-29) |  | 21 (13-30) |
| Padoan et al. 2020 | ** | 64 (47-71) |  | 35 (8-37) |  |  |
| Vultaggio et al. 2020 | * | 39 (30-51) | 23 (20-41) | 29 (8-36) |  | 16 (7-31) |
| Özdel Öztürk et al. 2022 | * | 37.32 (4.56) |  | 22.83 (3.10) |  | 23.56 (3.33) |

- 1. FEV1: Trends of FEV1 of the included patients over time after anti IL5 therapy initiation. FEV1 is expressed in percentage of the predicted value. *: cohort of patients treated with mepolizumab, **: cohort of patients treated with benralizumab

| **Study** | **treatment** | **0w** | **12w** | **24w** | **36w** | **48w** |
| --- | --- | --- | --- | --- | --- | --- |
| Bettiol et al. 2023 | ** | . | 90 (75-98) | 91 (72-98) |  | 94 (81-99) |
| Cottu et al. 2023 | ** | 74 (64-93) |  | 68 (55-90) |  | 93 (67-104) |
| Kim et al. 2010 | * | 79 (60-101) | 76 (49-102) |  |  |  |
| Nanzer et al. 2024 | ** | 73.9 (18.3) |  |  |  | 84.7 (21.1) |
| Nolasko et al. 2023 | ** | 73 (59-87) | 92 (70-101) | 88 (73-95) |  | 90 (70-101) |
|  | * | 80 (59-91) | 80 (69-99) | 83 (73-102) |  | 88 (71-103) |
| Özdel Öztürk et al. 2022 | * | 69.25 (4.77) |  | 75.61 (5.36) |  | 81.86 (7.03) |

1.6: Results of the Cochrane Collaboration’s tool for assessing risk of bias in randomized trials (RoB-2)


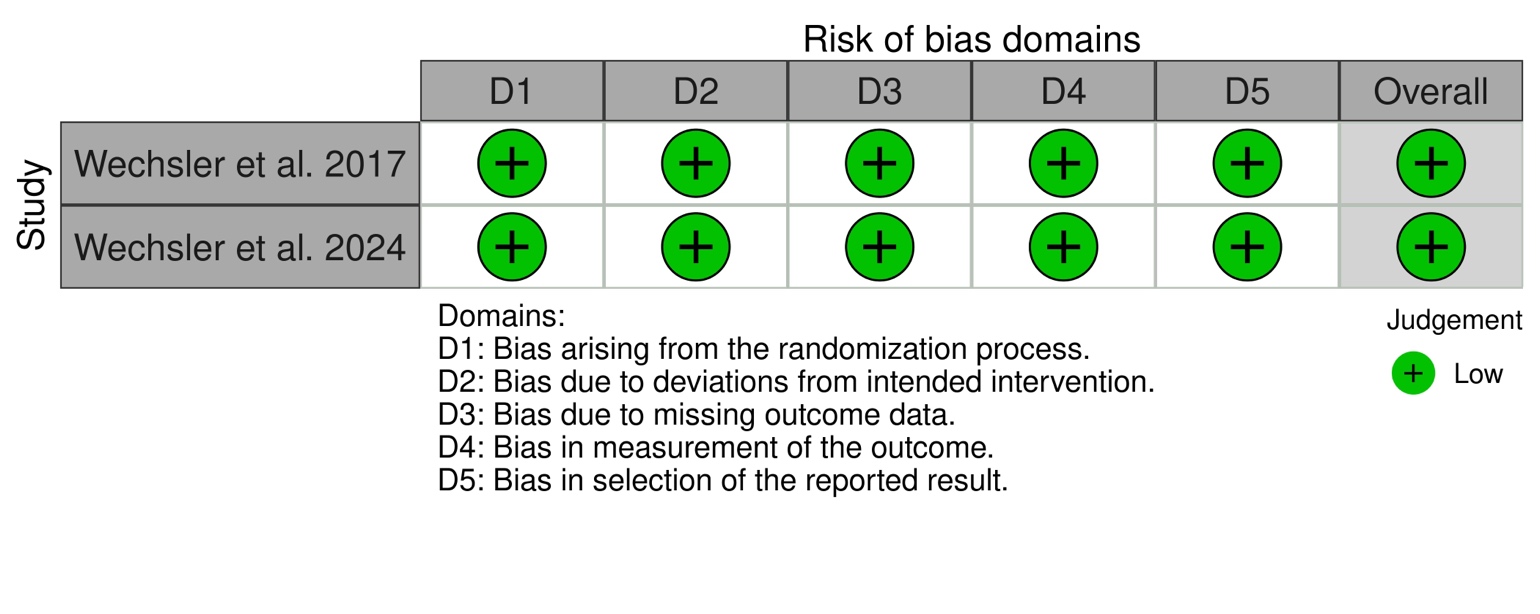


1.7 Results of the Risk Of Bias In Non randomized Studies of Interventions (ROBINS-I) tool for assessing risk of bias in observational studies


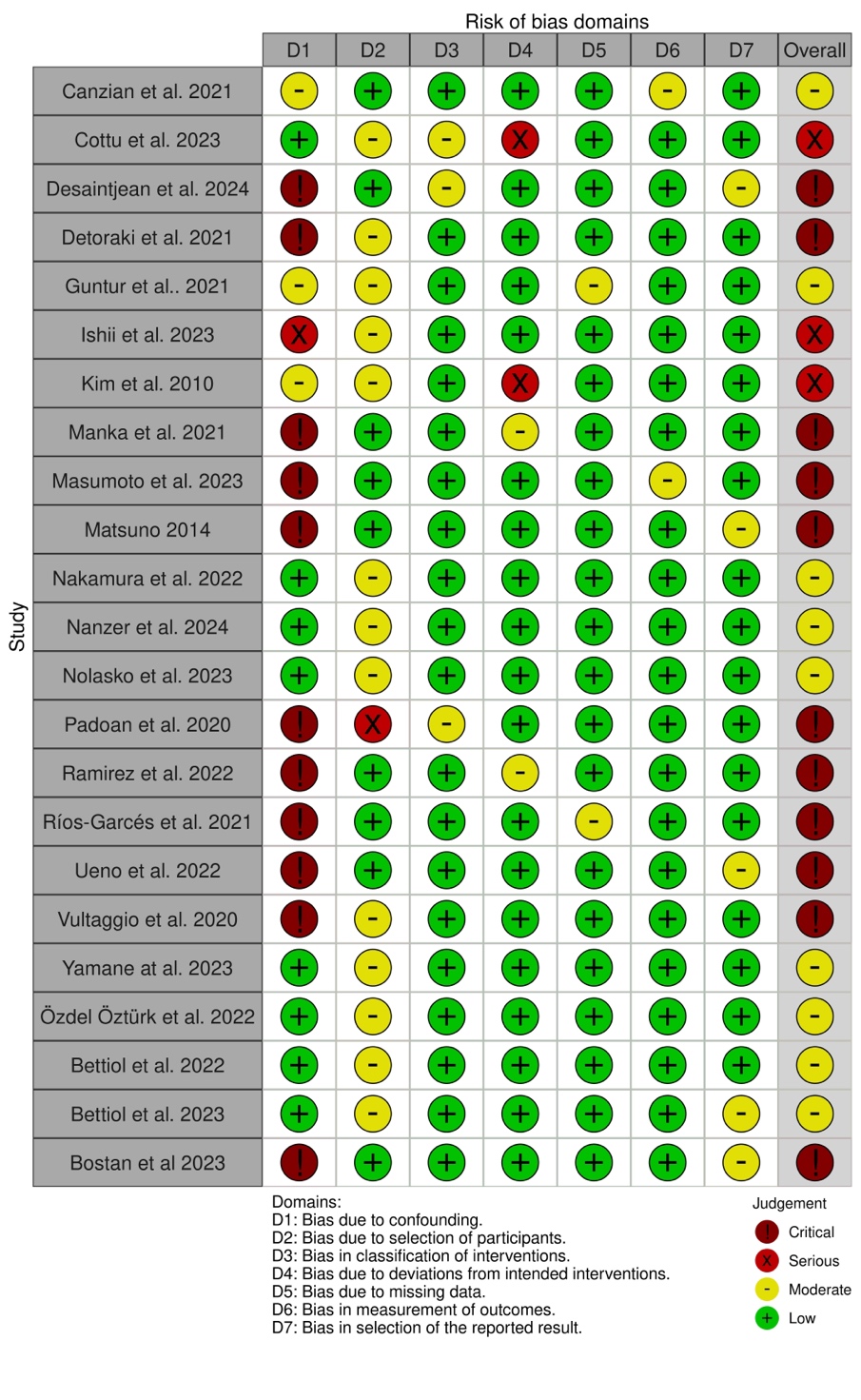


- 1. search strategies:

PubMed

((Churg-Strauss Syndrome[MeSH Terms]) OR “Churg-Strauss” OR “Churg Strauss” OR EGPA OR (Eosinophilic Granulomatosis with Polyangiit*[Title/Abstract])) AND (Mepolizumab OR Benralizumab OR “anti-IL-5 biologics” OR “anti-IL5”)

Embase

((Churg-Strauss Syndrome) OR “Churg-Strauss” OR “Churg Strauss” OR EGPA OR (Eosinophilic Granulomatosis with Polyangiitis)) AND (Mepolizumab OR Benralizumab OR “anti-IL-5 biologics” OR “anti-IL5”)

Cochrane library

((Churg-Strauss Syndrome) OR “Churg-Strauss” OR “Churg Strauss” OR EGPA OR (Eosinophilic Granulomatosis with Polyangiitis)) AND (Mepolizumab OR Benralizumab OR “anti-IL-5 biologics” OR “anti-IL5”)
